# A Cysteinyl-tRNA Synthetase Mutation Causes Novel Autosomal-Dominant Inheritance of a Parkinsonism/Spinocerebellar-Ataxia Complex

**DOI:** 10.1007/s12264-024-01231-0

**Published:** 2024-06-13

**Authors:** Han-Kui Liu, Hong-Lin Hao, Hui You, Feng Feng, Xiu-Hong Qi, Xiao-Yan Huang, Bo Hou, Chang-Geng Tian, Han Wang, Huan-Ming Yang, Jian Wang, Rui Wu, Hui Fang, Jiang-Ning Zhou, Jian-Guo Zhang, Zhen-Xin Zhang

**Affiliations:** 1 BGI Genomics and BGI Research, Shenzhen, 518083 China; 2 Department of Neurology, Clinical Epidemiology Unit, Peking Union Medical College Hospital, Chinese Academy of Medical Sciences, Beijing, 100730 China; 3 Division of Life Sciences and Medicine, University of Science and Technology of China, Hefei, 230026 China; 4 Department of Radiology, Peking Union Medical College Hospital, Chinese Academy of Medical Sciences, Beijing, 100730 China; 5 Department of Pathology, Beijing Key Laboratory of Biomarker Research and Transformation for Neurodegenerative Diseases, Peking University Third Hospital, School of Basic Medical Sciences, Peking University Health Science Center, Beijing, 100191 China; 6 Anhui Provincial Children’s Hospital, Children’s Hospital of Fudan University, Hefei, 230051 China; 7 Hebei Industrial Technology Research Institute of Genomics in Maternal and Child Health, Clin Lab, BGI Genomics, Shijiazhuang, 050011 China; 8 Institute of Brain Science, The First Affiliated Hospital of Anhui Medical University, Hefei, 230022 China

**Keywords:** Parkinsonism, Spinocerebellar ataxia, *CARS* gene

## Abstract

This study aimed to identify possible pathogenic genes in a 90-member family with a rare combination of multiple neurodegenerative disease phenotypes, which has not been depicted by the known neurodegenerative disease. We performed physical and neurological examinations with International Rating Scales to assess signs of ataxia, Parkinsonism, and cognitive function, as well as brain magnetic resonance imaging scans with seven sequences. We searched for co-segregations of abnormal repeat-expansion loci, pathogenic variants in known spinocerebellar ataxia-related genes, and novel rare mutations via whole-genome sequencing and linkage analysis. A rare co-segregating missense mutation in the *CARS* gene was validated by Sanger sequencing and the aminoacylation activity of mutant *CARS* was measured by spectrophotometric assay. This pedigree presented novel late-onset core characteristics including cerebellar ataxia, Parkinsonism, and pyramidal signs in all nine affected members. Brain magnetic resonance imaging showed cerebellar/pons atrophy, pontine-midline linear hyperintensity, decreased rCBF in the bilateral basal ganglia and cerebellar dentate nucleus, and hypo-intensities of the cerebellar dentate nuclei, basal ganglia, mesencephalic red nuclei, and substantia nigra, all of which suggested neurodegeneration. Whole-genome sequencing identified a novel pathogenic heterozygous mutation (E795V) in the *CARS* gene, meanwhile, exhibited none of the known repeat-expansions or point mutations in pathogenic genes. Remarkably, this *CARS* mutation causes a 20% decrease in aminoacylation activity to charge tRNA^Cys^ with L-cysteine in protein synthesis compared with that of the wild type. All family members carrying a heterozygous mutation *CARS* (E795V) had the same clinical manifestations and neuropathological changes of Parkinsonism and spinocerebellar-ataxia. These findings identify novel pathogenesis of Parkinsonism-spinocerebellar ataxia and provide insights into its genetic architecture.

**Supplementary Information:**

The online version contains supplementary material available at 10.1007/s12264-024-01231-0.

## Introduction

The autosomal dominant spinocerebellar ataxias (SCAs) are a clinically, pathologically, and genetically heterogeneous group of neurodegenerative disorders principally characterized by progressive cerebellar ataxia [1]. SCAs usually onset in middle age and present with extensive phenotypic variations that overlap with other motor/sensory neuropathology [2], such as parkinsonism, dystonia, chorea, and myoclonus [3], revealing more than 40 different phenotypic subtypes of SCAs [4]. Movement disorders are frequent in many of the various SCA subtypes and are even predominantly expressed in some SCA subtypes [3]. When combined with cerebellar ataxia, the occurrence of multisystem atrophy (MSA-C) makes differential clinical diagnoses particularly challenging. Most SCAs are caused by dynamic DNA-repeat expansions in specific genes [5], whereas other SCA subtypes are caused by point mutations [6]. Although the SCA subtypes exhibit complex phenotypic overlap, each SCA subtype corresponds to a specific causative gene [5, 6], indicating that genetic factors play a crucial role in the development of these subtypes. Here, we characterized a comprehensive spectrum of disease phenotypes in a 90-member Han Chinese family and identified a heterozygous mutation in cysteinyl-tRNA synthetase (*CARS*; E795V mutation) that significantly co-segregates in this pedigree. Moreover, the functional impact of mutation in *CARS* aminoacylation activity was further elucidated.

## Methods

### Participants and Clinical Evaluations

The first proband (IV-15) was evaluated at the Memory and Movement Disorder Center of Peking Union Medical College Hospital (PUMCH). In her family with a pedigree composed of 90 people from six generations, 17 individuals developed symptoms similar to those of the proband, while six died of illness and two refused any further examinations. Surveys were conducted at either a local hospital or within each individual’s home. Three professors of neurology collected the family and medical histories of each individual, performed physical and neurological examinations, and assessed signs of ataxia via the International Cooperative Ataxia Rating Scale (ICARS), signs of parkinsonism via the Unified Parkinson's Disease Rating Scale (UPDRS) part III, and cognitive function via a PUMCH-FHS neuropsychological test battery (Fig. S1). Brain magnetic resonance imaging (MRI) scans, via a GE Signa 3·0-T MRI scanner at PUMCH, were performed in four members with symptoms and in one member at the presymptomatic stage. MRI sequences consisted of sagittal three-dimensional (3D), T1-weighted, axial T2-weighted (1·0-mm isotropy), T2*-weighted, fluid-attenuated inversion recovery (FLAIR), susceptibility-weighted imaging (SWI), and arterial spin-labeling (ASL) images. Moreover, MRI scans of the neck and thoracic spinal cord, as well as electrophysiological examinations (i.e., sensor and motor nerve conduction velocities, somatosensory-evoked potentials, and auditory-evoked potentials), were also conducted for the first proband (IV-15).

### Whole-Genome Sequencing and Variants Calling

Whole-genome sequencing was used for identifying genetic variants, including single nucleotide variants (SNVs), short insertions-deletions (INDELs), short tandem repeats (STRs), copy number variants (CNVs), and structure variants (SVs). An average depth coverage of 66 × raw sequencing reads was generated by the BGISEQ-500 platform. Reads were filtered by SOAPnuke [7] and aligned to the human reference genome (hg19) by Burrows-Wheeler aligner [8] (version 0.7.15). Aligned reads were stored in cram format and the alignment summaries were calculated by samtools [9] (Table S1).

SNVs and INDELs were detected by the Genome Analysis Tool Kit [10] (version 3.6). Variants with any of the following quality control criticisms were excluded: (1) read depth < 4. (2) map quality < 55; (3) variant quality < 30; (4) genotype quality < 30. SNVs and INDELs were annotated by VEP [11] to predict the functional impact. Minor allele frequency (MAF) of variants were accessed in the gnomAD [12] database and two additional Chinese databases: ChinaMAP [13] and PGG.Han [14]. Variants with MAF > 1% in gnomAD [12] were excluded. The MAF cutoff threshold for rare variants was set at 1% by considering that deleterious alleles causing aging-related diseases may not be purged from the population before onset age. Patients with pathogenic mutation can survive for a long time and pass the mutant allele to their offspring, resulting in a low frequency and/or rare variant in the population.

Repeat-expansion calling was performed by expansionHunter [15]. The chromosomal locations, repeat motifs, diseases, and normal repeat-expansion sizes for 11 repeat-expansion loci of SCAs [5, 6, 16, 17] are listed in Table S2. A recently reported [18] repeat-expansion locus of SCA27B (*FGF14* [GAA]) was validated by Sanger sequencing. CNVs calling was performed by CNVnator [19]. CNVs with any of the quality control criticisms suggested by the author were excluded: (1) Q-value > 0.05; (2) fraction of reads with zero map quality > 0.5; (3) fraction of gap > 0. CNVs coordinates of all samples with 1 bp overlap were merged together by bedtools [20]. SVs calling was performed by LUMPY [21] (v.0.2.13) and SVs genotyping was performed by SVTyper [22] (v.0.1.4). SVs with any of the quality control criticisms employed from Abel’s study [23] were excluded: (1) The proportion of split-read and paired-end read counts < 10%; (2) mean sample quality < 150; (3) the deletion size < the insert size of sequencing library estimated by SVTyper; (4) deletion copy-number estimated by CNVnator [19] > 0.5 or duplication copy-number < 1.5.

### Linkage Analysis and Bioinformatic Analysis

To identify the pathological mutation, we performed linkage analysis via Merlin [24] software. We found three novel rare mutations co-segregated with the disease phenotype of sequencing individuals (four affected and three unaffected members) and no co-segregations of repeat-expansion, CNVs, SVs, and known pathogenic point mutations of SCA, Parkinson's disease [25, 26] (PD) and Multiple system atrophy [27] (MSA) reported by ClinVar database [28]. Sanger sequencing was used to validate the three mutations and confirm the co-segregations in the pedigree (all nine affected and seven unaffected members). Full-likelihood Bayes factor (BF) of co-segregation was calculated by segregated R-package to quantify the pathogenic evidence [29]. SIFT [30], PolyPhen-2 [31], M-CAP [32], and MutationTaster [33] were used to predict the functional impact of the mutations. Homologous sequence analysis was used to investigate the conservation of the *CARS* mutation across multiple species. I-TASSER [34] was used to model the structural consequence of the *CARS* protein altered by mutation.

### *CARS* Expression in Mouse Brain and Spinal Cord

To indicate whether the *CARS* gene is dosage sensitive, we employed a method developed in our previous study [35]. We collected 37 aminoacyl-tRNA synthetase genes in the human genome and calculated the standard deviation (SD) of gene expression across 156,049 mononuclear transcriptomes of mice brains and spinal cords (GSE110823) [36] via the formula: $SD=\sqrt{\frac{\sum_{i}^{n}{({C}_{i}-\overline{C })}^{2}}{n-1}}$, in which $C$ refers to fold change, $i$ refers to the $i$ th cell, and $n$ refers to the total number of cells. The fold change was calculated by the expression in one cell divided by the mean expression of all cells: ${C}_{i}={E}_{i}/\overline{E }$, in which $E$ refers to the expression in one cell, $\overline{E }$ refers to the mean expression, $i$ refers to the $i$ th cell. We compared the SD of the *CARS* gene with loss-of-function tolerant genes [37] and dosage-sensitive genes [38]. Mann-Whitney U-test was used to indicate the significance of low SD values of aminoacyl-tRNA synthetase genes compared with loss-of-function tolerant genes and dosage-sensitive genes.

### *CARS* Immunohistochemistry

Paraffin-embedded temporal cortex sections (obtained from Netherlands Brain Bank) from a 92-year-old control male were hydrated, rinsed in phosphate-buffered saline (PBS) for 10 min, and treated with 1.5% hydrogen peroxide in PBS for 1 hour at 37℃ to quench endogenous peroxidase activity. After being washed in PBS containing 0.5% triton X-100 (PBST) (3*5 min), the sections were treated with microwaves (700 W) in 0.05 mol/L citrate-buffered saline (pH 6.0) for 2×10 min for antigen retrieval. Subsequently, the sections were washed in PBST (3×5 min) and incubated in 5% normal goat serum (Vector Laboratories, Burlingame, CA) in PBST for 1 hour at 37℃ to block nonspecific staining. Then the sections were incubated with primary antiserum of rabbit anti-*CARS* (Novus biological, NBP1-86624, at 1:200 dilution) in PBST containing 5% normal goat serum for 24 hours at 4℃. Amplification was performed with biotinylated goat anti-rabbit IgG (1:200; Vector Laboratories, USA) and avidin-biotin-peroxidase complex (1:200; Vector Laboratories). Finally, the immune complex was visualized by incubation with 0.05% 3, 3’-diaminobenzidine (Sigma-Aldrich) in PBST containing 0.03% H2O2 for 10 min. The sections were mounted in Tris–HCl buffer containing 0.5% gelatin and dried overnight at room temperature. Photographs were collected using an Olympus BX52 microscope (Olympus, Japan).

### Aminoacylation Activity of Mutant *CARS*

Quantification of aminoacylation of tRNA is used to measure the ability of mutant *CARS* to catalyze the attachment of L-cystine to tRNA^cys^ (Fig. S5). Wild-type (WT) *CARS* and a known mutant *CARS* (c.2061) that have been reported to modify the aminoacylation activity [39] were used for negative and positive control. A high throughput spectrophotometric assay for quantitative measurement is designed and performed in a transparent 96-well plate [40]. Each 100μl aminoacylation reaction containing 30 mmol/L HEPES (pH7.5), 30 mmol/L NaCl, 30 mmol/L KCl, 40 mmol/L MgCl2, 1 mmol/L DTT, 200 μmol/L ATP, 2 U/mL PPiase, 55 μg/mL miRNA and enzyme was incubated at 37℃ for an hour. In aminoacylation, pyrophosphate was released in ATP catabolism, and catalyzed to inorganic phosphate by PPiase. EDTA was used to terminate the aminoacylation and a 25 μL malachite green dye was added to each reaction and then the reaction was incubated at room temperature for 10 minutes. After that, the absorbance value of the chromogenic complex of inorganic phosphorus and malachite green dye was measured by a multimode microplate reader at 620 nm and its standard curve was estimated.

## Results

### Clinical Characteristics

**Fig. 1:**
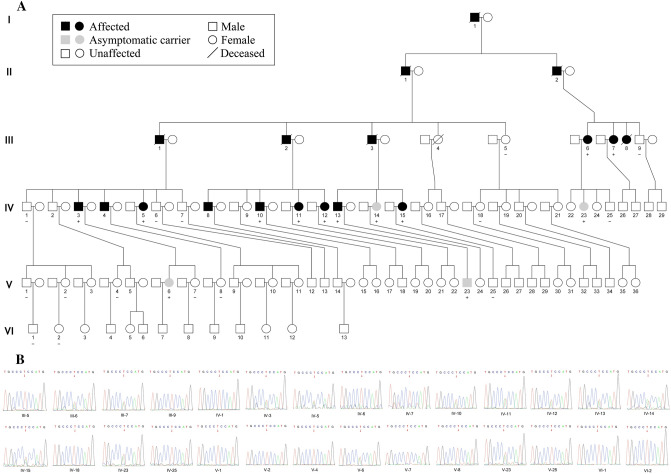
A heterozygous *CARS* mutation causes a rare neurodegenerative disease in a 90-member family. The first proband (IV-15) was evaluated at the Memory and Movement Disorder Center at Peking Union Medical College Hospital (PUMCH). Three neurologists examined all 30 available participants in this pedigree and identified nine affected members and 21 clinically unaffected members. Four affected members (III-7, IV-5, IV-12, and IV-15) and three age-matched unaffected members (III-5, III-9, and IV-1) were used for whole-genome sequencing. A significant co-segregated rare mutation, *CARS*: p.Glu795Val, was identified in whole-genome-sequencing samples and was confirmed, via Sanger sequencing, in five additional affected members (III-6, 75 years old; IV-4, 63 years old; IV-10, 52 years old; IV-11, 50 years old; IV-13, 52 years old) and four additional unaffected members who were at or close to the onset age of disease (IV-6, 52 years old; IV-7, 48 years old; IV-18, 52 years old; IV-25, 38 years old). All 30 members (except for IV-26 and V-24 with failure of Sanger sequencing) with available DNA are marked with (+) or without (–) the *CARS* mutation **(A)**, and their genotypes are marked by the red arrows in the *CARS* sequence chromatograms **(B)**.

**Fig. 2:**
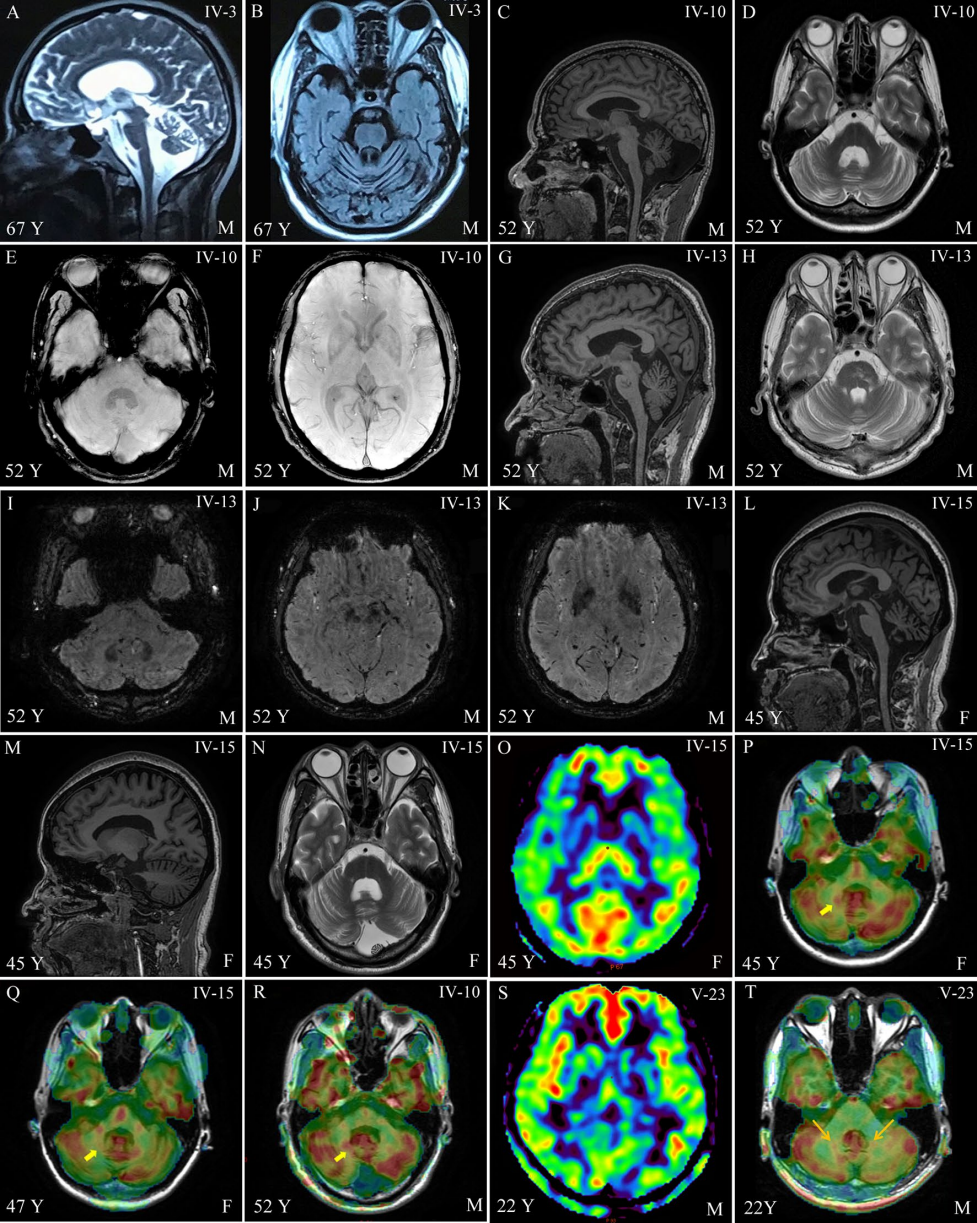
Magnetic resonance imaging in the brain. Brain MRI scans were performed on four clinically affected family members (IV-3, IV-10, IV-13, and, IV-15), and one asymptomatic member carrying the *CARS* mutation (IV-23). **A** Marked atrophy of the pons, vermis, and **B** cerebellar cortex is shown in MRI scans from member IV-3; **C** Atrophy of the pons, vermis, and **D** cerebellar cortex with a pontine-midline linear hyperintensity on T2WI is shown for member IV-10. **E, F** A normal iron-induced SWI hypo-signal in the dentate nuclei and basal ganglia is shown in MRI scans from member IV-10. **G, H** Mild atrophy of the pons, vermis, and cerebellar cortex with lacunas in the pons is shown in MRI scans from member IV-13. **I–K** An iron-induced SWI hypo-intensity in the dentate nuclei, red nuclei, SN, and basal ganglia is shown in MRI scans from member IV-13.** L** Marked atrophy of the vermis, pons, and **L–N** diffuse cerebellar atrophy with **N** a pontine-midline linear hyperintensity on T2WI is shown in MRI scans from member IV-15. **O** Decreased regional cerebral blood flow (rCBF) in the bilateral basal ganglia (from IV-15) and **P** in the cerebellar dentate nucleus, **Q** with further deterioration over two years, is shown in MRI scans from member IV-15. **R** Decreased rCBF in the cerebellar dentate nucleus (from member IV-10). **S** Decreased rCBF in the left putamen and the head of the caudate nucleus (from V-23), **T** but not in the cerebellar dentate nucleus.

Among all 30 available participants, nine affected members were identified from four families of offspring in the third or fourth generation of this pedigree (Fig. 1A). The specific clinical features are shown in Table 1. The onset age ranged from 42–62 years and the duration of the disease varied from 1–25 years. All nine affected members presented core characteristics, namely difficulty in walking as an early feature, slow progression, followed by varying degrees of cerebellar ataxia, parkinsonism, and pyramidal signs (except IV-12, one-year course, no pyramidal signs). The severities of these three core clinical manifestations were closely related to the course of the disease. All patients over the eight-year course were confined to wheelchairs. The non-core features consisted of a variety of neurodegenerative manifestations. Among them, peripheral neuropathy and vertical gaze dysfunction were only found in the posterity's family D (III-6, III-7). Cognitive impairment, stridor, and cold hands were only found in the posterity's family C (IV-3, IV-5). Erectile dysfunction was found only in the posterity's family B (IV-13), and facial grimacing was found in the posterity's families A and C (IV-5, IV-12, IV-10). Four of the nine affected members underwent brain MRI scans, and all showed varying degrees of atrophy in the cerebellar cortex, vermis, and pons (Table 1, Fig. 2, Fig. S2). The axial T2-weighted images showed pontine-midline linear hyperintensities, similar to MSA-C in two affected members (IV-10, IV-15). SWI revealed hypo-intensities of the cerebellar dentate nuclei, basal ganglia, mesencephalic red nuclei, and substantia nigra (SN) in one affected member (IV-10), suggesting iron accumulation. ASL images showed decreased regional cerebral blood flow (rCBF) in the bilateral basal ganglia and the cerebellar dentate nucleus, followed by further deterioration over two years (IV-15), whereas there was reduction only in the dentate nuclei for the member at the pre-symptomatic stage (IV-10). Moreover, the following were all normal in the first proband (IV-15): cervical and thoracic spinal cord MRI scans; electrophysiological examinations of sensor- and motor-nerve conduction velocities; somatosensory-evoked potentials; and auditory-evoked potentials. These findings excluded cervical and thoracic spinal cord demyelination, peripheral neuropathy, deep sensory disorder, and auditory nerve damage.

**Table 1:** Clinical phenotypes

Generation	Posterity's family A (III-2)			Posterity's family B (III-3)		Posterity's family C (III-1)		Posterity's family D (II-2)	
	IV-12	IV-11	IV-10	IV-13	IV-15	IV-5	IV-3	III-6	III-7
Age at the exam (years)	47	52	52	52	45	60	67	75	70
Sex	F	F	M	M	F	F	M	F	F
Age at onset (years)	46	50	46	51	42	52	55	62	45
Disease Duration (years)	1	2	6	1	3	8	12	13	25
The first symptom at the onset	Unable to run	Slow down the stairs	Unable to run	Unable to run	Walking unstable	Walks with knees bent	Walking unstable	Walking unstable	Walking unstable
Gait ataxia*	1	1	3	1	2	8	8	8	8
Nystagmus	Horizontal	No	No	No	Horizontal	No	No	No	Horizontal
Oculomotor deficit	No	No	No	No	No	No	Abduction	No	Vertical
Dysmetria*	No	1 in left	1	1 in left	1	3	3	2	2
Dysarthria*	1	No	1	No	1	4	3	1	1
Tremor	No	No	Action	No	No	No	No	No	No
Bradykinesia**	1	2	2	2	2	3	2	2	2
Rigidity**	No	1 in left	2	2	1	1	1	3	3
Others related signs	No	No	No	No	No	Stridor/Cold hands	No	No	No
Reflex	++	+++ left	+++	++++	+++	++++	++++	Achilles +	Achilles 0
Pathologic signs	No	No	Babinski	Babinski	Hoffman	Babinski	Babinski	Babinski	Babinski
Bucking - bulbar signs	No	Bucking	Bucking	No	Bucking	Bucking	Bucking	Bucking	Bucking
Emotional lability	No	No	Lability	No	No	Lability	No	No	No
Urinary dysfunction	No	No	No	No	Urgency	Urgency	Incontinent	No	Incontinent
Erectile dysfunction	NA	NA	No	+	NA	NA	No	NA	NA
Blood pressure while sitting & standing	125/95 120/90	130/85 130/90	135/100 135/90	183/105 180/120	130/90 115/85	Normal	Normal	170/110 200/124	140/100 170/110
Peripheral Neuropathy	No	No	No	No	No	No	No	Lower limbs	Lower limbs
Dystonia	Right hand	No	Facial grimacing	No	No	Facial grimacing	No	No	No
Cognitive function^§^	Normal	Normal	Normal	Normal	Normal	Dementia	Dementia	Normal	Normal
MMSE	29	28	27	25	26	10	12	22^#^	21^#^
PUMCH-FHS NP battery^§^	NA	NA	Normal	NA	Normal	NA	NA	NA	NA
*Brain MRI*									
Atrophy of cerebellar/pons	NA	NA	Mild	Mild	Moderate	NA	Severe	NA	NA
Iron-induced hypointensity	NA	NA	Normal	Absent	Obvious	NA	NA	NA	NA
Pontine-midline linear hyperintensity	NA	NA	Present	Suspicious	Hot cross bun	NA	NA	NA	NA
Decreased rCBF‡	NA	NA	Dentate N	No	BG, Dentate N	NA	NA	NA	NA
Cervical & thoracic Spinal Cord MRI	NA	NA	NA	NA	Normal	NA	NA	NA	NA
Electrophysiological examination	NA	NA	NA	NA	Normal	NA	NA	NA	NA

*ICAR score; **UPDRS Part-III; ^§^PUMCH-FHS Neuropsychological assessment battery consisted of 13 tests in memory, executive functions, language, visual-spatial abilities, and attention for IV -10 and IV-15 patients; DSM-5 criteria are used for diagnosis of dementia; ^#^an illiterate person; ‡dentate N: cerebellar dentate nucleus; BG: basal ganglia.

### Pathogenic Variant Identification

To identify the genetic cause of disease in this pedigree, we selected four affected members (III-7, IV-5, IV-12, and IV-15) and three age-matched unaffected members (III-5, 79 years old; III-9, 65 years old; IV-1, 72 years old) to perform whole-genome sequencing (Table S1). All seven members were identified to exhibit normal ranges of expression levels in 11 repeat-expansion loci of SCAs (Table S2) and did not carry any reported pathogenic mutations (SNVs and INDELs) related to SCAs, indicating that known pathogenic mutations were not the cause of disease in this pedigree. The normal repeat number of two loci (SCA17: *TBP*[CAG]; SCA37: *DAB1*[ATTTC]) caused similar phenotypes and a recently reported locus (SCA27B: *FGF14*[GAA]) was validated by Sanger sequencing in the proband (IV-15) (Fig. S3). To identify the novel genetic cause, we screened all the variants—including SNVs, INDELs, CNVs, and SVs—and then found three rare missense mutations (Table 2) co-segregated in this pedigree (Merlin: LOD=2.9). We performed Sanger sequencing for the three candidate mutations in all 30 members with available DNA (Fig. 1B). A *CARS* heterozygous mutation (c.2384A>T; p.Glu795Val; E795V) was detected in all nine affected members, whereas no such mutation was detected in any of the seven unaffected members who were at or close to the onset-age of disease (III-5, 79 years old; III-9, 65 years old; IV-1, 72 years old; IV-6, 52 years old; IV-7, 48 years old; IV-18, 52 years old; IV-25, 38 years old). *ERVFRD-1* mutation and *OR51G2* mutation were not detected in affected member IV-13 (Fig. S4). We calculate the LOD score and BF of co-segregation in nine affected and seven unaffected members via Merlin and segregated R-package. Notably, a LOD score of 4.96 indicated the *CARS* mutation is significantly co-segregated with the disease status in this pedigree. A BF score of 4631 is strong evidence that suggests *CARS* mutation is pathogenicity. We screened the SVs and CNVs in the E795V neighboring region and found none of the SVs/CNVs at the *CARS* gene, which is consistent with the rare event of *CARS* SVs/CNVs in the gnomAD v4 database. These results indicated *CARS* E795V is the cause of the disease.

**Table 2:** Candidate mutations

Gene	ID	Mutation	Minor Allele Frequency			Pathogenicity Prediction			
			gnomAD	ChinaMAP	PGG.Han	SIFT	Polyphen2	M-CAP	MutationTaster
*CARS*	rs139920420	c.2384A>T; p.Glu795Val	0.0008	0	0.006	D	P	D	D
*ERVFRD-1*	rs138651238	c.1565C>T; p.Thr522Met	0.00199	0.021	0.023	T	T	-	T
*OR51G2*	rs150667862	c.907C>G; p.Gln303Glu	0.0002	0.004	0.003	T	T	T	D

“-” indicates the mutation was unpredictable. gnomAD refers to the gnomAD database. ChinaMAP refers to a Chinese deep whole-genome sequencing database of 10,588 individuals. PGG.Han refers to the Han-Chinese genetic variants database of 126,590 individuals. The pathogenicity of the mutated allele was predicted as D(damaging), P(possibly damaging), and T(tolerated) by SIFT, Polyphen2, M-CAP, and MutationTaster, respectively.

Specifically, four currently unaffected members (IV-14, 45 years old; IV-23, 46 years old; V-6, 42 years old; V-23, 22 years old) carried this pathogenic mutation. V-23 had undertaken brain MRI scans and showed preclinical features, but the other three unaffected members refused to undergo MRIs or further examinations.

### Functional Impact

**Fig. 3:**
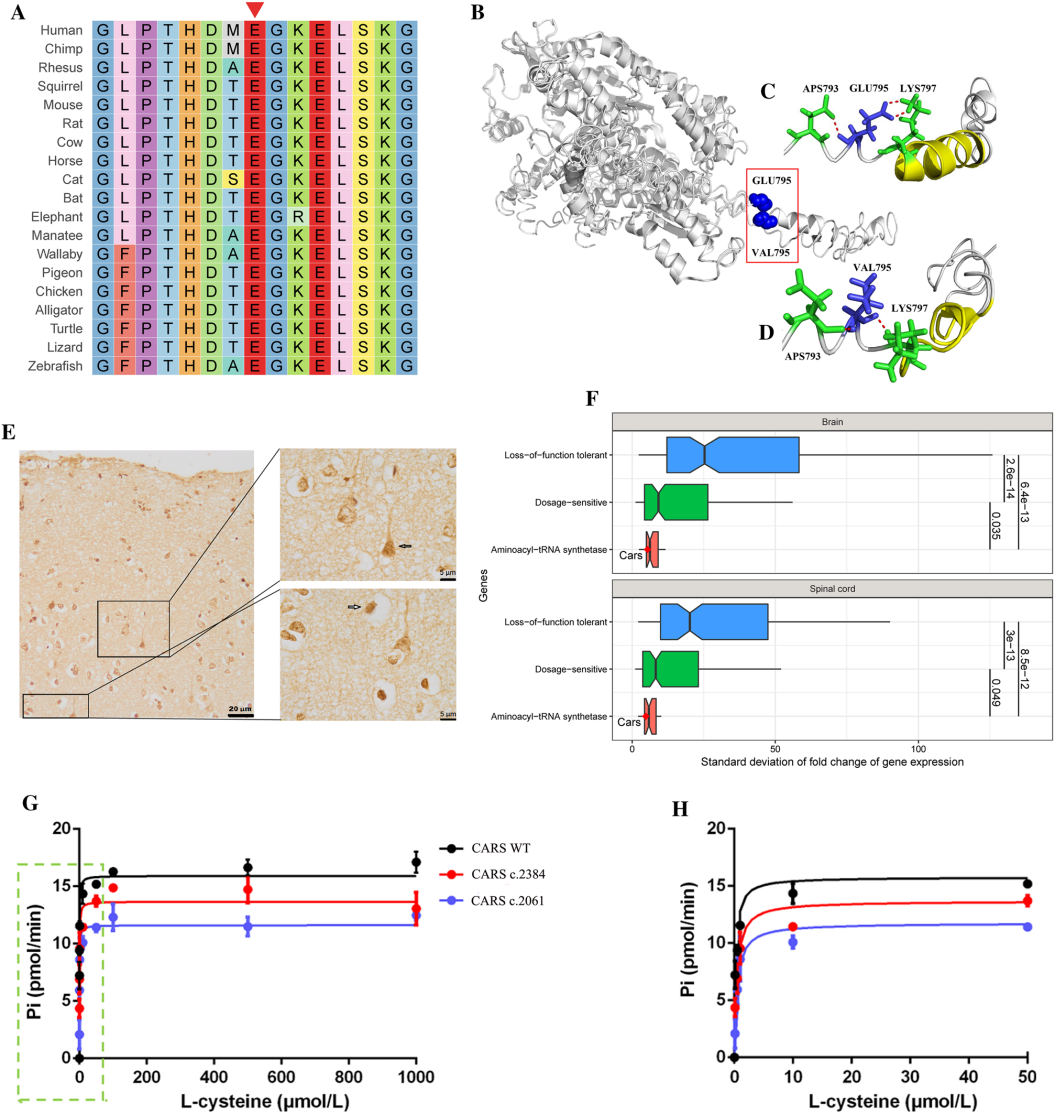
The impact of *CARS* mutation.** A** Evolutionary conservation. The red triangle indicates that the mutated amino acid at codon-795 of CARS orthologs is highly conserved. **B–D** Prediction of 3D protein structure. The human CARS protein structure is displayed as a ball-and-stick model **B**. Compared with the wild type **C**, the mutated variant **D** destroyed one of three backbone hydrogen bonds and changed the α-helix to a loop.** E** *CARS* expression in human brain cortex. Paraffin-embedded temporal-cortex sections (Netherlands Brain Bank) from a 92-year-old control male were stained with the *CARS* antibody (Novus Biological, NBP1-86624). **F** The strict requirement of *CARS* expression in mice brain and spinal cord. The standard deviation (SD) of fold change of gene expression is used to evaluate the spectrum of expression variation. A low SD value indicates a gene requires strict expression. Dosage-sensitive genes are known to be intolerant of expression alteration and present lower SD values. Loss-of-function tolerant genes are known to be tolerant of expression alteration and present higher SD values. The significance of Mann-Whitney U-tests exhibits that *CARS* (redpoint) expression is strictly required in mice brain/spinal cord, indicating *CARS* expression is intolerant to be altered. **G–H** The aminoacylation activity of *CARS in vitro*. Curves in a green rectangle of** G** were detailed in **H**. The curves of the initial rate (pmol/min) at different concentrations of L-cysteine are fitted by the Michaelis-Menten formula. Compared with WT, a 20% decrease in the activity of mutant *CARS* was estimated by the fitted curves. The differences in curves between WT and a mutant *CARS* (c.2061) known to reduce activity demonstrate our experiment results are reliable.

*CARS* mutation is novel (0) in the Chinese population of the ChinaMAP database, and rare (0.006) in Han-Chinese of PGG. Han database and (0.008) in East Asian of gnomAD-v3 [12] database (Table 2). This rare mutation was predicted to have a harmful effect on gene function (Table 2), be conservative across multi-species (Fig. 3A), and predicted to induce a conformational change to protein structure (Fig. 3B-D), and *CARS* were found to be expressed in the cytoplasm of neurons within the human temporal cortex (Fig. 3E). Moreover, single-cell transcriptome analysis showed that the standard deviation (SD) of *Cars* expression fold change is significantly low compared with that of dosage-sensitive genes (Fig. 3F), indicating *Cars* expression is strictly required in normal brain/spinal cord and its expression alteration is intolerant. More importantly, we observed a decreased aminoacylation activity of mutant *CARS*, as well as a known mutant *CARS* (c.2061) that has been reported to reduce the aminoacylation activity [41] via spectrophotometric assay *in vitro* (Fig. 3G-H)*.* The fitted curve of the initial rate estimated a 20% decrease in the activity of mutant *CARS* compared with that of *CARS* WT (Table 3). These results indicated that *CARS* mutation altered the molecular dynamics of WT *CARS* and caused a decreased activity to charge tRNA^Cys^ with L-cysteine in protein synthesis. Taken together with bioinformatic prediction, statistical evidence, and molecular evidence *in vitro*, we suggested that *CARS* mutation is the genetic cause of disease.

**Table 3:** The dynamics characteristic of enzyme activity of mutant *CARS* gene

Characteristics	*CARS* WT	*CARS* c.2384	*CARS* c.2061
Vmax (pmol/min)	15.91	13.66	11.61
Km (μmol/L)	0.3394	0.3792	0.3888
Kcat (s-1)	0.0071	0.0061	0.0052
Activity compared to wild-type	1	0.77	0.64

*CARS* WT, *CARS* c.2384, and *CARS* c.2061 refer to wild-type *CARS*, mutant *CARS* in our study, and a *CARS* mutation known to reduce aminoacylation activity.

## Discussion

In the present study, we discovered a novel late-onset parkinsonism/SCA complex in a 90-member family, which presented core characteristics in all nine affected members. The affected members in this family displayed a variety of other clinical features consistent with those of rare neurodegenerative diseases, such as vertical gaze palsies, facial grimacing, cognitive impairment, peripheral neuropathy, autonomic dysfunction, blood-vessel dysfunction, and bladder dysfunction, as well as rare symptoms such as erectile dysfunction, stridor, and cold hands. Interestingly, posterity's families exhibited specific non-core clinical manifestations. The brain MRI findings in all four available members suggested that loci of pathological changes were not limited to the cerebellum and brainstem but were also present in the basal ganglia, mesencephalic red nucleus, and the SN. The pathological characteristics not only consisted of atrophy and a “hot cross bun” sign in the pons but also included abnormal iron deposition and a reduction of rCBF. These findings suggest that this disorder is a novel rare neurodegenerative disease, namely a parkinsonism/SCA complex. However, these clinical manifestations may exist in different SCA subtypes. SCAs—particularly SCA2, SCA3, SCA6, SCA8, and SCA17—can exhibit parkinsonism [42]. The “hot cross bun” sign in the pons of axial T2-weighted images may occur in certain SCA subtypes (SCA1, SCA2, SCA3, SCA6, SCA7, SCA17, SCA8, and SCA34) despite being more a common feature MSA [43–45]. Additionally, most of these SCAs present a shorter expansion of CAG repeats. However, none of these SCA subtypes can solely represent the diverse clinical manifestations of affected members in the pedigree that we analyzed in the present study. In addition, impaired horizontal eye movement has been reported in many SCA subtypes, whereas abnormal vertical eye movement is a typical sign of progressive supranuclear palsy, which is a rare symptom and generally presents as a relatively pure cerebellar phenotype in SCA37 [46]. In our present study, the affected member (III-7) with vertical gaze palsies and other neurodegenerative signs cannot be considered as having a subtype of SCA37. Although gait ataxia was the first symptom of all affected family members in the present study, a 22-year-old asymptomatic carrier (V-23) showed reduced rCBF only in the left putamen and caudate nuclei, but not in the dentate nuclei, which has not been reported for any SCA subtype [47]. Furthermore, an affected member (IV-10) had iron accumulation in the basal ganglia, mesencephalic red nucleus, SN, and cerebellar dentate nucleus, which in combination with other neurodegenerative signs, should be considered a rare neurodegenerative disease rather than SCA17 [48]. Additionally, dynamic DNA-repeat expansions in 12 SCA loci were identified to be within normal ranges and none of the pathogenic point mutations found in the pedigree of the present study were related to SCAs, PD, or MSA; hence, these results did not provide any genetic evidence for known SCA subtypes. Moreover, the clinical manifestations found in the present pedigree were different from those of MSA. MSA generally progresses rapidly. Early and severe autonomic nerve failure is a major feature of most MSA patients, with 78.3% having orthostatic hypotension, 86.9% having urinary incontinence, and 94.3% having erectile dysfunction. To the best of our knowledge, no previous study has reported such extensive, rich, and specific clinical and neuropathological manifestations of affected members within such a large pedigree [46, 49–54], which cannot be explained by other known disorders or by the functions of genes implicated in these disorders [55–57].

In the genetic analysis, we identified a *CARS* missense mutation as the pathogenic variant in this large domain pedigree. *CARS*, namely *CARS1*, is responsible for charging tRNAs with cysteine during protein synthesis. *CARS* is a member of ARSs, in which each ARS is responsible for ligating a specific amino acid to its cognate tRNA during protein synthesis. Recent studies have shown that mutations in ARSs cause a variety of neurological disorders [58]. Many mutants result in loss of tRNA aminoacylation function [59].Aminoacylation experiment revealed that mutant *AARS* [60]*, AARS2* [61]*, MARS* [62]*, QARS* [63]*, VARS* [64]*, NARS1* [65], *KARS* [66], *HARS1* [67], *SARS* [68], and *CARS* [39] severely reduces enzymic activity. Kuo ME’s study showed that *CARS* bi-allele caused a multi-system, recessive disease that includes microcephaly, developmental delay, and brittle hair and nails [39]. The pathogenic variants R341H and S359L reduced 40% and 70% of *CARS* activity, respectively. In contrast, our study showed that the *CARS* E795V variant causes a 20% decreasing activity of aminoacylation in protein synthesis. *CARS* genes are dosage-sensitive in mice brains and spinal cords. The SD value of *CARS* gene expression is lower than the median of ARSs genes that presented significantly low SDs compared with dosage-sensitive genes (*P* < 8.5×10^-12^) or loss-of-function tolerant genes (*P* < 0.05). Loss-of-function tolerant genes are tolerant of expression alteration and present low SD values. Oppose to loss-of-function tolerant genes, dosage-sensitive genes are intolerant of expression alteration and present high SD values. These results suggested that the 20% decreasing activity of aminoacylation caused a loss of function effect on the *CARS* gene function. Although loss of function is the mechanism of diseases caused by *CARS* pathogenic variants, the reduction of *CARS* activity is differed by E795V(20%), R341H(40%), and S359L(70%) mutant. The R341H and S359L affect the catalytic domain that activates and transfers cysteine to tRNA. E795V was localized in the C-terminal extension, which is different from that of loss of function caused by R341H, S359L, and other ARS pathogenic variants. On the other hand, the loss of function of *DARS* [69], *RARS* [70], *KARS* [66], *HARS1* [67], *SARS* [68]*,* and *NARS2*/*PARS2* [71] were reported to cause neurological diseases that share ataxic feature, indicating an important role of ARS function in the pathogenesis of ataxia. *HARS1* pathogenic variant caused a multisystem ataxic syndrome. *DARS*, *RARS*, *KARS*, and *AARS2* pathogenic variants caused ataxia with or without leukoencephalopathy. *SARS* pathogenic variant caused complex spastic paraplegia with microcephaly, intellectual disability, and ataxia. *NARS2* and *PARS2* pathogenic variants caused Alpers–Huttenlocher syndrome with ataxia. Our study of the *CARS* pathogenic variant expands the genetic spectrum of ARS-related ataxia.

In summary, we reported that a *CARS* pathogenic variant (E795V) is associated with a domain parkinsonism/SCA presentation. These results may broaden the spectrum of human diseases and provide insight into the genetic architecture of ARS-related neurological disorders.

## Supplementary Information

Below is the link to the electronic supplementary material.

Supplementary file1 (PDF 3112 KB)

Supplementary file2 (XLSX 15 KB)

## Data Availability

The necessary code was released on GitHub (https://github.com/liuhankui/WGS-linkage). Analyses were performed using public software packages: quality control of genome sequence (SOAPnuke, https://github.com/BGI-flexlab/SOAPnuke); alignment (BWA, https://github.com/lh3/bwa); variant calling of SNV and INDEL (GATK, https://github.com/broadinstitute/gatk), repeat-expansion (ExpansionHunter, https://github.com/Illumina/ExpansionHunter), CNV (CNVnator, https://github.com/abyzovlab/CNVnator), and SV (LUMPY, https://github.com/arq5x/lumpy-sv; SVtyper, https://github.com/hall-lab/svtyper); variant annotation (VEP, https://github.com/Ensembl/ensembl-vep); linkage analysis (MERLIN, http://csg.sph.umich.edu/abecasis/merlin/index.html); co-segregation analysis (https://github.com/magnusdv/segregatr); protein structure prediction (I-TASSER, https://zhanglab.ccmb.med.umich.edu/I-TASSER); data visualization (R, https://www.r-project.org).

## References

[1] Dueñas AM, Goold R, Giunti P. Molecular pathogenesis of spinocerebellar ataxias. Brain 2006, 129: 1357–1370. 16613893 10.1093/brain/awl081

[2] Schöls L, Peters S, Szymanski S, Krüger R, Lange S, Hardt C. Extrapyramidal motor signs in degenerative ataxias. Arch Neurol 2000, 57: 1495–1500. 11030803 10.1001/archneur.57.10.1495

[3] van Gaalen J, Giunti P, van de Warrenburg BP. Movement disorders in spinocerebellar ataxias. Mov Disord 2011, 26: 792–800. 21370272 10.1002/mds.23584

[4] Ashizawa T, Öz G, Paulson HL. Spinocerebellar ataxias: Prospects and challenges for therapy development. Nat Rev Neurol 2018, 14: 590–605. 30131520 10.1038/s41582-018-0051-6 PMC6469934

[5] Durr A. Autosomal dominant cerebellar ataxias: Polyglutamine expansions and beyond. Lancet Neurol 2010, 9: 885–894. 20723845 10.1016/S1474-4422(10)70183-6

[6] Schöls L, Bauer P, Schmidt T, Schulte T, Riess O. Autosomal dominant cerebellar ataxias: Clinical features, genetics, and pathogenesis. Lancet Neurol 2004, 3: 291–304. 15099544 10.1016/S1474-4422(04)00737-9

[7] Chen Y, Chen Y, Shi C, Huang Z, Zhang Y, Li S, *et al*. SOAPnuke: A MapReduce acceleration-supported software for integrated quality control and preprocessing of high-throughput sequencing data. Gigascience 2018, 7: gix120. 29220494 10.1093/gigascience/gix120 PMC5788068

[8] Li H, Durbin R. Fast and accurate short read alignment with Burrows-Wheeler transform. Bioinformatics 2009, 25: 1754–1760. 19451168 10.1093/bioinformatics/btp324 PMC2705234

[9] Danecek P, Bonfield JK, Liddle J, Marshall J, Ohan V, Pollard MO, *et al*. Twelve years of SAMtools and BCFtools. GigaScience 2021, 10: giab008. 33590861 10.1093/gigascience/giab008 PMC7931819

[10] McKenna A, Hanna M, Banks E, Sivachenko A, Cibulskis K, Kernytsky A, *et al*. The Genome Analysis Toolkit: A MapReduce framework for analyzing next-generation DNA sequencing data. Genome Res 2010, 20: 1297–1303. 20644199 10.1101/gr.107524.110 PMC2928508

[11] McLaren W, Pritchard B, Rios D, Chen Y, Flicek P, Cunningham F. Deriving the consequences of genomic variants with the Ensembl API and SNP Effect Predictor. Bioinformatics 2010, 26: 2069–2070. 20562413 10.1093/bioinformatics/btq330 PMC2916720

[12] Karczewski KJ, Francioli LC, Tiao G, Cummings BB, Alföldi J, Wang Q, *et al*. The mutational constraint spectrum quantified from variation in 141, 456 humans. Nature 2020, 581: 434–443. 32461654 10.1038/s41586-020-2308-7 PMC7334197

[13] Cao Y, Li L, Xu M, Feng Z, Sun X, Lu J, *et al*. The ChinaMAP analytics of deep whole genome sequences in 10, 588 individuals. Cell Res 2020, 30: 717–731. 32355288 10.1038/s41422-020-0322-9 PMC7609296

[14] Gao Y, Zhang C, Yuan L, Ling Y, Wang X, Liu C, *et al*. PGG.Han: The Han Chinese genome database and analysis platform. Nucleic Acids Res 2020, 48: D971–D976. 31584086 10.1093/nar/gkz829 PMC6943055

[15] Dolzhenko E, Deshpande V, Schlesinger F, Krusche P, Petrovski R, Chen S, *et al*. ExpansionHunter: A sequence-graph-based tool to analyze variation in short tandem repeat regions. Bioinformatics 2019, 35: 4754–4756. 31134279 10.1093/bioinformatics/btz431 PMC6853681

[16] Shakkottai VG, Fogel BL. Clinical neurogenetics: Autosomal dominant spinocerebellar ataxia. Neurol Clin 2013, 31: 987–1007. 24176420 10.1016/j.ncl.2013.04.006 PMC3818725

[17] Tankard RM, Bennett MF, Degorski P, Delatycki MB, Lockhart PJ, Bahlo M. Detecting expansions of tandem repeats in cohorts sequenced with short-read sequencing data. Am J Hum Genet 2018, 103: 858–873. 30503517 10.1016/j.ajhg.2018.10.015 PMC6288141

[18] Rafehi H, Read J, Szmulewicz DJ, Davies KC, Snell P, Fearnley LG, *et al*. An intronic GAA repeat expansion in FGF14 causes the autosomal-dominant adult-onset ataxia SCA27B/ATX-FGF14. Am J Hum Genet 2023, 110: 1018. 37267898 10.1016/j.ajhg.2023.05.005 PMC10257192

[19] Abyzov A, Urban AE, Snyder M, Gerstein M. CNVnator: An approach to discover, genotype, and characterize typical and atypical CNVs from family and population genome sequencing. Genome Res 2011, 21: 974–984. 21324876 10.1101/gr.114876.110 PMC3106330

[20] Quinlan AR, Hall IM. BEDTools: A flexible suite of utilities for comparing genomic features. Bioinformatics 2010, 26: 841–842. 20110278 10.1093/bioinformatics/btq033 PMC2832824

[21] Layer RM, Chiang C, Quinlan AR, Hall IM. LUMPY: A probabilistic framework for structural variant discovery. Genome Biol 2014, 15: R84. 24970577 10.1186/gb-2014-15-6-r84 PMC4197822

[22] Chiang C, Layer RM, Faust GG, Lindberg MR, Rose DB, Garrison EP, *et al*. SpeedSeq: Ultra-fast personal genome analysis and interpretation. Nat Methods 2015, 12: 966–968. 26258291 10.1038/nmeth.3505 PMC4589466

[23] Abel HJ, Larson DE, Regier AA, Chiang C, Das I, Kanchi KL, *et al*. Mapping and characterization of structural variation in 17, 795 human genomes. Nature 2020, 583: 83–89. 32460305 10.1038/s41586-020-2371-0 PMC7547914

[24] Abecasis GR, Cherny SS, Cookson WO, Cardon LR. Merlin—rapid analysis of dense genetic maps using sparse gene flow trees. Nat Genet 2002, 30: 97–101. 11731797 10.1038/ng786

[25] Blauwendraat C, Nalls MA, Singleton AB. The genetic architecture of Parkinson’s disease. Lancet Neurol 2020, 19: 170–178. 31521533 10.1016/S1474-4422(19)30287-X PMC8972299

[26] Liu Y, Niu L, Liu X, Cheng C, Le W. Recent progress in non-motor features of Parkinson’s disease with a focus on circadian rhythm dysregulation. Neurosci Bull 2021, 37: 1010–1024. 34128188 10.1007/s12264-021-00711-x PMC8275711

[27] Tseng FS, Foo JQX, Mai AS, Tan EK. The genetic basis of multiple system atrophy. J Transl Med 2023, 21: 104. 36765380 10.1186/s12967-023-03905-1 PMC9912584

[28] Landrum MJ, Chitipiralla S, Brown GR, Chen C, Gu B, Hart J, *et al*. ClinVar: Improvements to accessing data. Nucleic Acids Res 2020, 48: D835–D844. 31777943 10.1093/nar/gkz972 PMC6943040

[29] Jarvik GP, Browning BL. Consideration of cosegregation in the pathogenicity classification of genomic variants. Am J Hum Genet 2016, 98: 1077–1081. 27236918 10.1016/j.ajhg.2016.04.003 PMC4908147

[30] Kumar P, Henikoff S, Ng PC. Predicting the effects of coding non-synonymous variants on protein function using the SIFT algorithm. Nat Protoc 2009, 4: 1073–1081. 19561590 10.1038/nprot.2009.86

[31] Adzhubei IA, Schmidt S, Peshkin L, Ramensky VE, Gerasimova A, Bork P, *et al*. A method and server for predicting damaging missense mutations. Nat Methods 2010, 7: 248–249. 20354512 10.1038/nmeth0410-248 PMC2855889

[32] Jagadeesh KA, Wenger AM, Berger MJ, Guturu H, Stenson PD, Cooper DN, *et al*. M-CAP eliminates a majority of variants of uncertain significance in clinical exomes at high sensitivity. Nat Genet 2016, 48: 1581–1586. 27776117 10.1038/ng.3703

[33] Schwarz JM, Rödelsperger C, Schuelke M, Seelow D. MutationTaster evaluates disease-causing potential of sequence alterations. Nat Methods 2010, 7: 575–576. 20676075 10.1038/nmeth0810-575

[34] Roy A, Kucukural A, Zhang Y. I-TASSER: A unified platform for automated protein structure and function prediction. Nat Protoc 2010, 5: 725–738. 20360767 10.1038/nprot.2010.5 PMC2849174

[35] Liu H, Guan L, Deng M, Bolund L, Kristiansen K, Zhang J, *et al*. Integrative genetic and single cell RNA sequencing analysis provides new clues to the amyotrophic lateral sclerosis neurodegeneration. Front Neurosci 2023, 17: 1116087. 36875658 10.3389/fnins.2023.1116087 PMC9983639

[36] Rosenberg AB, Roco CM, Muscat RA, Kuchina A, Sample P, Yao Z, *et al*. Single-cell profiling of the developing mouse brain and spinal cord with split-pool barcoding. Science 2018, 360: 176–182. 29545511 10.1126/science.aam8999 PMC7643870

[37] Lek M, Karczewski KJ, Minikel EV, Samocha KE, Banks E, Fennell T, *et al*. Analysis of protein-coding genetic variation in 60, 706 humans. Nature 2016, 536: 285–291. 27535533 10.1038/nature19057 PMC5018207

[38] Dang VT, Kassahn KS, Marcos AE, Ragan MA. Identification of human haploinsufficient genes and their genomic proximity to segmental duplications. Eur J Hum Genet 2008, 16: 1350–1357. 18523451 10.1038/ejhg.2008.111

[39] Kuo ME, Theil AF, Kievit A, Malicdan MC, Introne WJ, Christian T, *et al*. Cysteinyl-tRNA synthetase mutations cause a multi-system, recessive disease that includes microcephaly, developmental delay, and brittle hair and nails. Am J Hum Genet 2019, 104: 520–529. 30824121 10.1016/j.ajhg.2019.01.006 PMC6407526

[40] Cestari I, Stuart K. A spectrophotometric assay for quantitative measurement of aminoacyl-tRNA synthetase activity. J Biomol Screen 2013, 18: 490–497. 23134734 10.1177/1087057112465980 PMC3774597

[41] Goel P, Parvez S, Sharma A. Genomic analyses of aminoacyl tRNA synthetases from human-infecting helminths. BMC Genomics 2019, 20: 333. 31046663 10.1186/s12864-019-5679-0 PMC6498573

[42] Park H, Kim HJ, Jeon BS. Parkinsonism in spinocerebellar ataxia. Biomed Res Int 2015, 2015: 125273. 25866756 10.1155/2015/125273 PMC4383270

[43] Ozaki K, Doi H, Mitsui J, Sato N, Iikuni Y, Majima T, *et al*. A novel mutation in ELOVL4 leading to spinocerebellar *Ataxia* (SCA) with the hot cross bun sign but lacking erythrokeratodermia: A broadened spectrum of SCA34. JAMA Neurol 2015, 72: 797–805. 26010696 10.1001/jamaneurol.2015.0610

[44] Heidelberg D, Ronsin S, Bonneville F, Hannoun S, Tilikete C, Cotton F. Main inherited neurodegenerative cerebellar ataxias, how to recognize them using magnetic resonance imaging? J Neuroradiol 2018, 45: 265–275. 29920348 10.1016/j.neurad.2018.05.005

[45] Way C, Pettersson D, Hiller A. The ‘hot cross bun’ sign is not always multiple system atrophy: Etiologies of 11 cases. J Mov Disord 2019, 12: 27–30. 30563313 10.14802/jmd.18031 PMC6369380

[46] Serrano-Munuera C, Corral-Juan M, Stevanin G, San Nicolás H, Roig C, Corral J, *et al*. New subtype of spinocerebellar ataxia with altered vertical eye movements mapping to chromosome 1p32. JAMA Neurol 2013, 70: 764–771. 23700170 10.1001/jamaneurol.2013.2311

[47] Xing W, Wang XY, Liao XX, Liao WH, Shen L. Spin labeling artery method perfusion MRI study of SPG4 and SCA3/MJD. Magn Reson Imaging 2014, 32: 1330–1334. 25172988 10.1016/j.mri.2014.08.022

[48] Claassen J, Gerding WM, Kastrup O, Uslar E, Goericke S, Timmann D. Excessive brain iron accumulation in spinocerebellar ataxia type 17. Neurology 2015, 84: 212–213. 25583826 10.1212/WNL.0000000000001141

[49] Shimazaki H, Vazifehmand R, Heidari MH, Khorram-Khorshid HR, Saber S, Hejazi S, *et al*. A large family with spinocerebellar ataxia type 6 in Iran: A clinical and genetic study. Arch Iran Med 2008, 11: 459–462. 18588381

[50] Rosa AL, Molina I, Kowaljow V, Conde CB. Brisk deep-tendon reflexes as a distinctive phenotype in an Argentinean spinocerebellar ataxia type 2 pedigree. Mov Disord 2006, 21: 66–68. 16108012 10.1002/mds.20636

[51] Wang JL, Yang X, Xia K, Hu ZM, Weng L, Jin X, *et al*. TGM6 identified as a novel causative gene of spinocerebellar ataxias using exome sequencing. Brain 2010, 133: 3510–3518. 21106500 10.1093/brain/awq323

[52] Sun H, Satake W, Zhang C, Nagai Y, Tian Y, Fu S, *et al*. Genetic and clinical analysis in a Chinese parkinsonism-predominant spinocerebellar ataxia type 2 family. J Hum Genet 2011, 56: 330–334. 21307863 10.1038/jhg.2011.14

[53] Cadieux-Dion M, Turcotte-Gauthier M, Noreau A, Martin C, Meloche C, Gravel M, *et al*. Expanding the clinical phenotype associated with ELOVL4 mutation: Study of a large French-Canadian family with autosomal dominant spinocerebellar ataxia and erythrokeratodermia. JAMA Neurol 2014, 71: 470–475. 24566826 10.1001/jamaneurol.2013.6337

[54] Wang C, Xu Y, Feng X, Ma J, Xie S, Zhang Y, *et al*. Linkage analysis and whole-exome sequencing exclude extra mutations responsible for the parkinsonian phenotype of spinocerebellar ataxia-2. Neurobiol Aging 2015, 36(545): e1-545.e7. 25189117 10.1016/j.neurobiolaging.2014.07.039

[55] Harding AE. Classification of the hereditary ataxias and paraplegias. Lancet 1983, 1: 1151–1155. 6133167 10.1016/s0140-6736(83)92879-9

[56] Seidel K, Siswanto S, Brunt ERP, den Dunnen W, Korf HW, Rüb U. Brain pathology of spinocerebellar ataxias. Acta Neuropathol 2012, 124: 1–21. 22684686 10.1007/s00401-012-1000-x

[57] Rossi M, Perez-Lloret S, Doldan L, Cerquetti D, Balej J, Millar Vernetti P, *et al*. Autosomal dominant cerebellar ataxias: A systematic review of clinical features. Eur J Neurol 2014, 21: 607–615. 24765663 10.1111/ene.12350

[58] Boczonadi V, Jennings MJ, Horvath R. The role of tRNA synthetases in neurological and neuromuscular disorders. FEBS Lett 2018, 592: 703–717. 29288497 10.1002/1873-3468.12962 PMC5873386

[59] Antonellis A, Green ED. The role of aminoacyl-tRNA synthetases in genetic diseases. Annu Rev Genomics Hum Genet 2008, 9: 87–107. 18767960 10.1146/annurev.genom.9.081307.164204

[60] Simons C, Griffin LB, Helman G, Golas G, Pizzino A, Bloom M, *et al*. Loss-of-function alanyl-tRNA synthetase mutations cause an autosomal-recessive early-onset epileptic encephalopathy with persistent myelination defect. Am J Hum Genet 2015, 96: 675–681. 25817015 10.1016/j.ajhg.2015.02.012 PMC4385183

[61] Kuo ME, Antonellis A, Shakkottai VG. Alanyl-tRNA synthetase 2 (AARS2)-related *Ataxia* without leukoencephalopathy. Cerebellum 2020, 19: 154–160. 31705293 10.1007/s12311-019-01080-y PMC6982554

[62] van Meel E, Wegner DJ, Cliften P, Willing MC, White FV, Kornfeld S, *et al*. Rare recessive loss-of-function methionyl-tRNA synthetase mutations presenting as a multi-organ phenotype. BMC Med Genet 2013, 14: 106. 24103465 10.1186/1471-2350-14-106 PMC3852179

[63] Zhang X, Ling J, Barcia G, Jing L, Wu J, Barry BJ, *et al*. Mutations in QARS, encoding glutaminyl-tRNA synthetase, cause progressive microcephaly, cerebral-cerebellar atrophy, and intractable seizures. Am J Hum Genet 2014, 94: 547–558. 24656866 10.1016/j.ajhg.2014.03.003 PMC3980424

[64] Stephen J, Nampoothiri S, Banerjee A, Tolman NJ, Penninger JM, Elling U, *et al*. Loss of function mutations in VARS encoding cytoplasmic valyl-tRNA synthetase cause microcephaly, seizures, and progressive cerebral atrophy. Hum Genet 2018, 137: 293–303. 29691655 10.1007/s00439-018-1882-3 PMC7251970

[65] Wang L, Li Z, Sievert D, Smith DEC, Mendes MI, Chen DY, *et al*. Loss of NARS1 impairs progenitor proliferation in cortical brain organoids and leads to microcephaly. Nat Commun 2020, 11: 4038. 32788587 10.1038/s41467-020-17454-4 PMC7424529

[66] Sun C, Song J, Jiang Y, Zhao C, Lu J, Li Y, *et al*. Loss-of-function mutations in Lysyl-tRNA synthetase cause various leukoencephalopathy phenotypes. Neurol Genet 2019, 5: e565. 31192300 10.1212/NXG.0000000000000316 PMC6515944

[67] Galatolo D, Kuo ME, Mullen P, Meyer-Schuman R, Doccini S, Battini R, *et al*. Bi-allelic mutations in HARS1 severely impair histidyl-tRNA synthetase expression and enzymatic activity causing a novel multisystem ataxic syndrome. Hum Mutat 2020, 41: 1232–1237. 32333447 10.1002/humu.24024 PMC7323910

[68] Verdura E, Senger B, Raspall-Chaure M, Schlüter A, Launay N, Ruiz M, *et al*. Loss of seryl-tRNA synthetase (*SARS1*) causes complex spastic paraplegia and cellular senescence. J Med Genet 2022, 59: 1227–1233. 36041817 10.1136/jmg-2022-108529 PMC9691831

[69] Wolf NI, Toro C, Kister I, Latif KA, Leventer R, Pizzino A, *et al*. DARS-associated leukoencephalopathy can mimic a steroid-responsive neuroinflammatory disorder. Neurology 2015, 84: 226–230. 25527264 10.1212/WNL.0000000000001157 PMC4335995

[70] Nafisinia M, Sobreira N, Riley L, Gold W, Uhlenberg B, Weiß C, *et al*. Mutations in RARS cause a hypomyelination disorder akin to Pelizaeus-Merzbacher disease. Eur J Hum Genet 2017, 25: 1134–1141. 28905880 10.1038/ejhg.2017.119 PMC5602020

[71] Mizuguchi T, Nakashima M, Kato M, Yamada K, Okanishi T, Ekhilevitch N, *et al*. PARS2 and NARS2 mutations in infantile-onset neurodegenerative disorder. J Hum Genet 2017, 62: 525–529. 28077841 10.1038/jhg.2016.163

