# Supplementary material for: A Cysteinyl-tRNA Synthetase Mutation Causes Novel Autosomal-Dominant Inheritance of a Parkinsonism/Spinocerebellar-Ataxia Complex
Source: Neurosci Bull. 2024 Jun 13;40(10):1489–501. doi: 10.1007/s12264-024-01231-0 (PMC11422396; doi:10.1007/s12264-024-01231-0)
Supplement: Supplementary file 1 — Supplementary file1 (PDF 3112 KB) [file 12264_2024_1231_MOESM1_ESM.pdf]

Supplementary Figures Legends

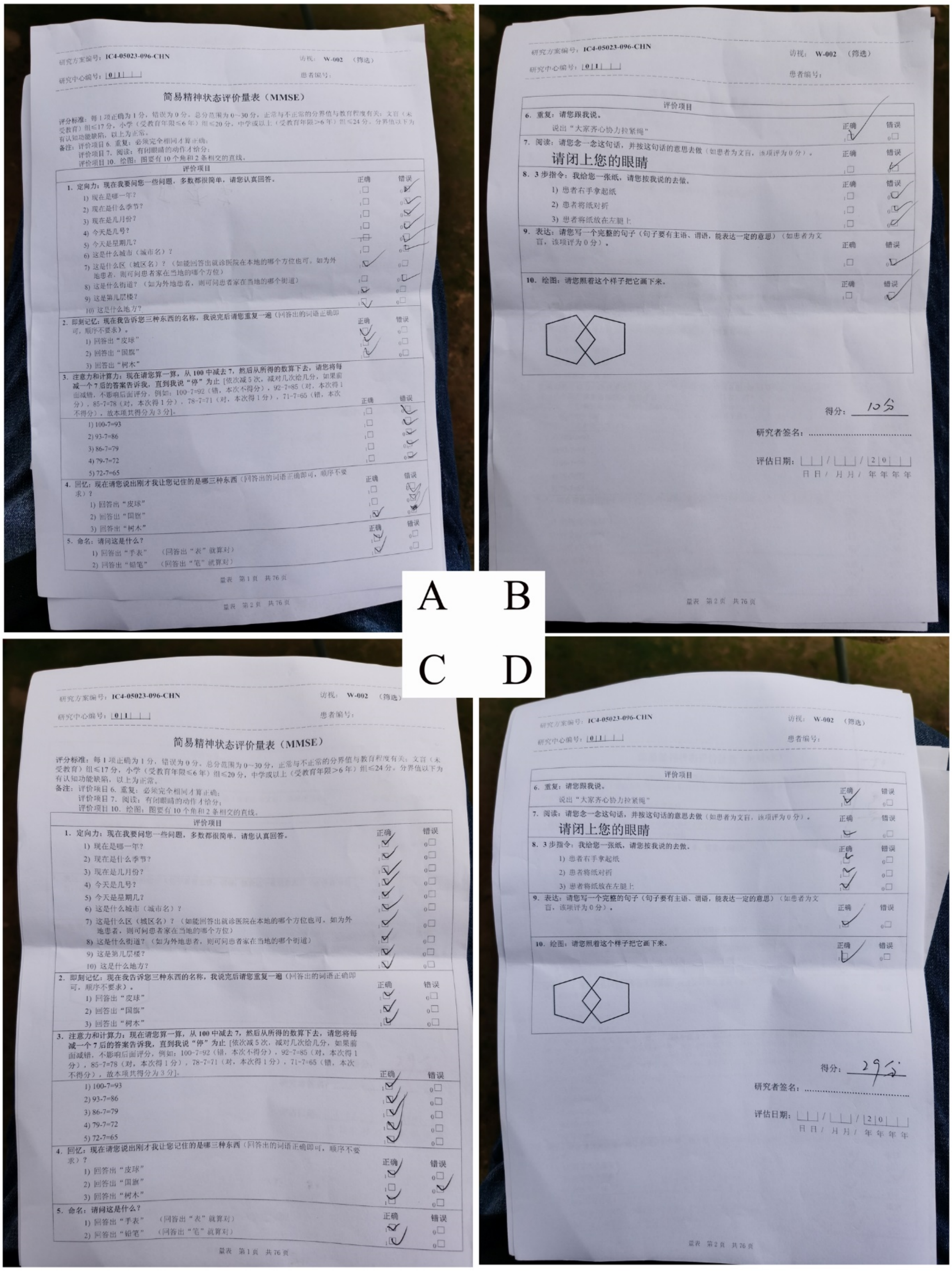

Fig. S1. PUMCH-FHS neuropsychological test battery. The cognitive function of IV-5 (**A, B**) and IV-15 (**C, D**) was examined by three professors of neurology.

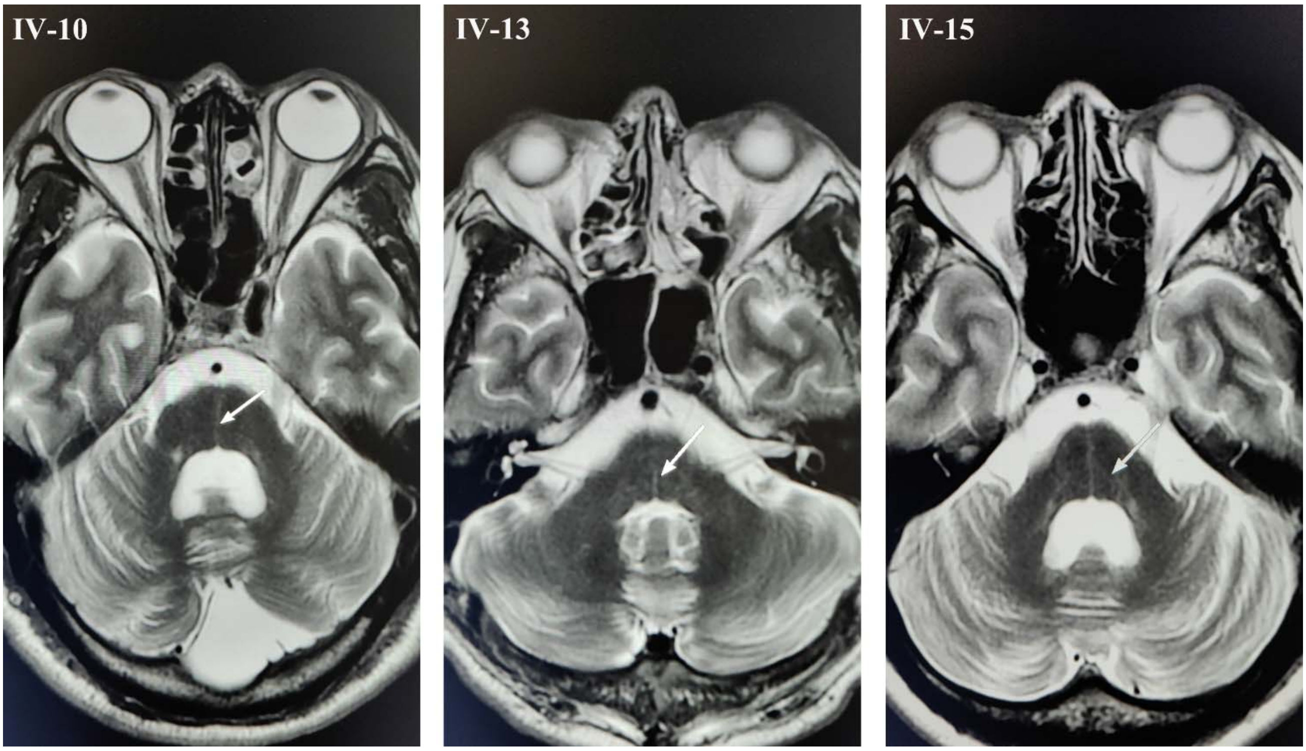

Fig. S2. A blowup picture of the pons.

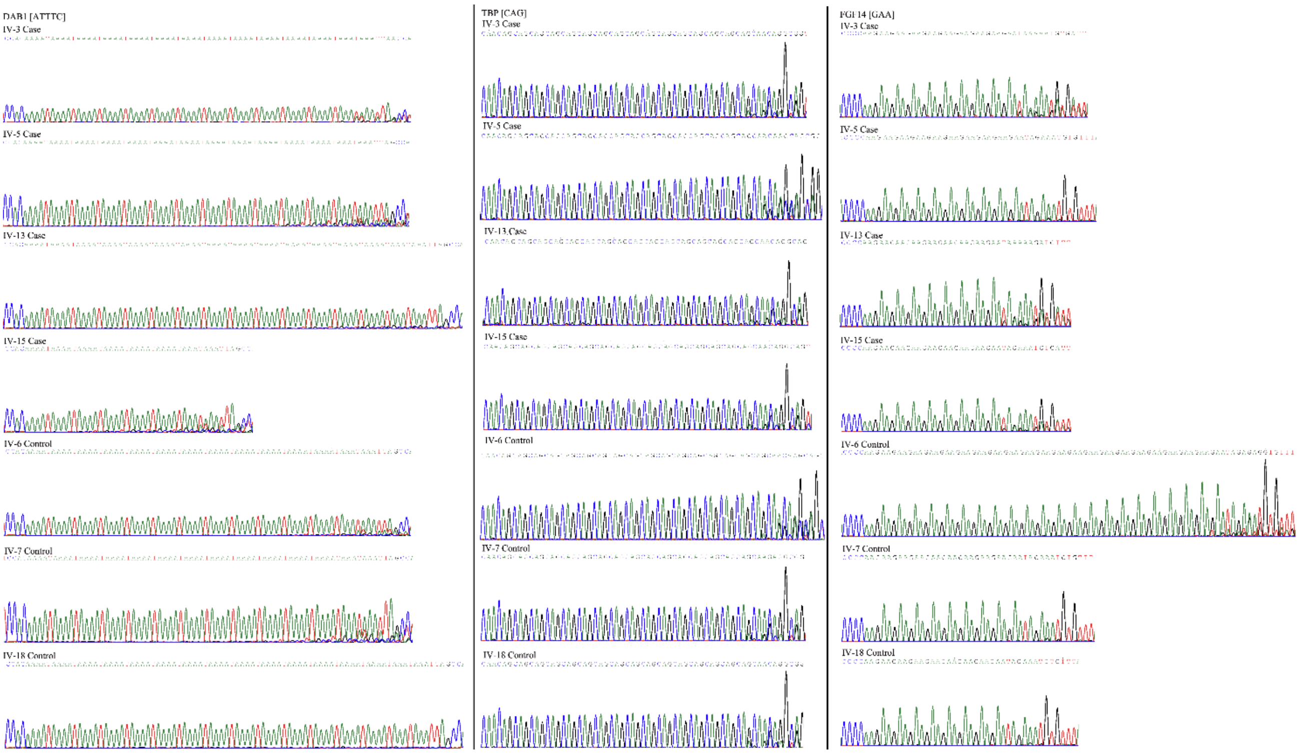

Fig. S3. Sanger sequencing for the *DAB1* gene ATTTC repeat unit, the *TBP* gene CAG repeat unit, and the *FGF14* gene GAA repeat unit. The proband IV-15 and other affected members harbored normal repeat numbers at the three genes.

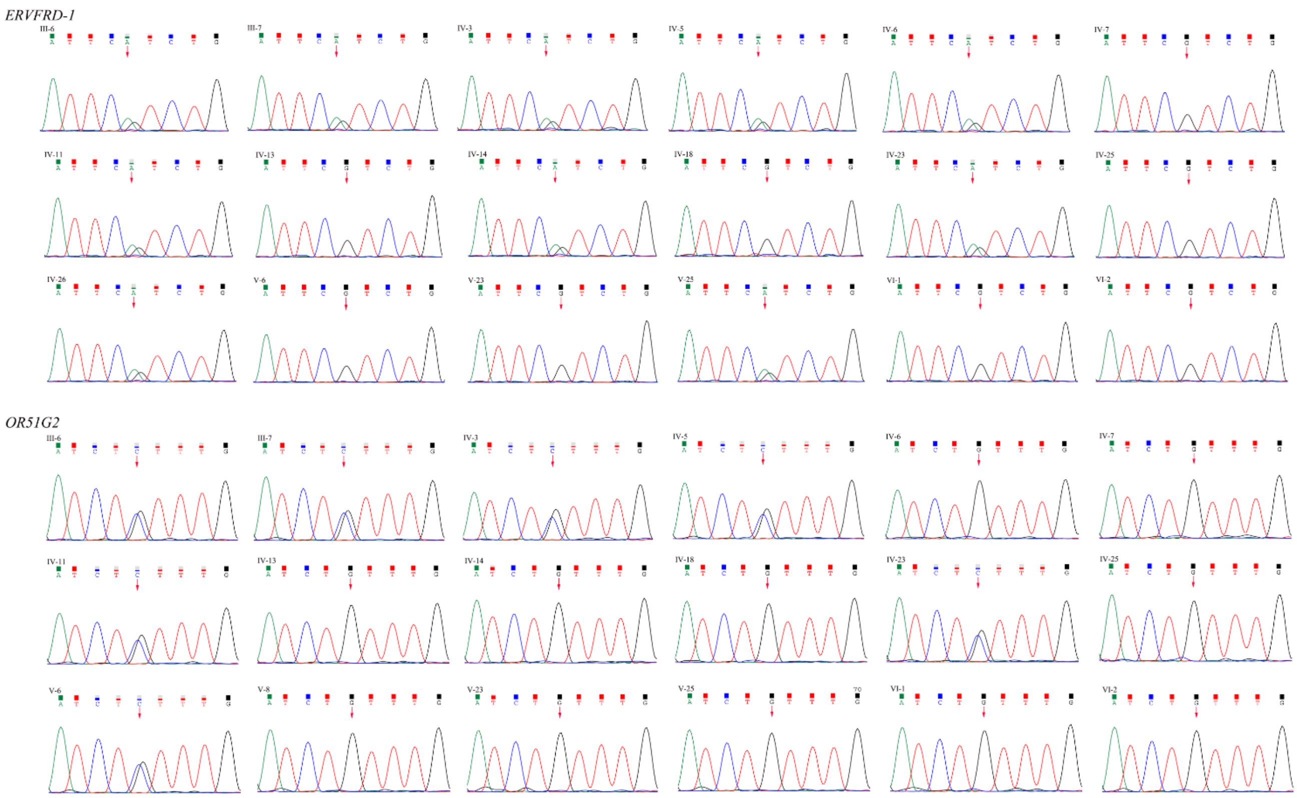

Fig. S4. Sanger sequencing for *ERVFRD-1* mutation and *OR51G2* mutation. The affected member IV-13 does not carry the *ERVFRD-1* mutation nor *OR51G2* mutation.

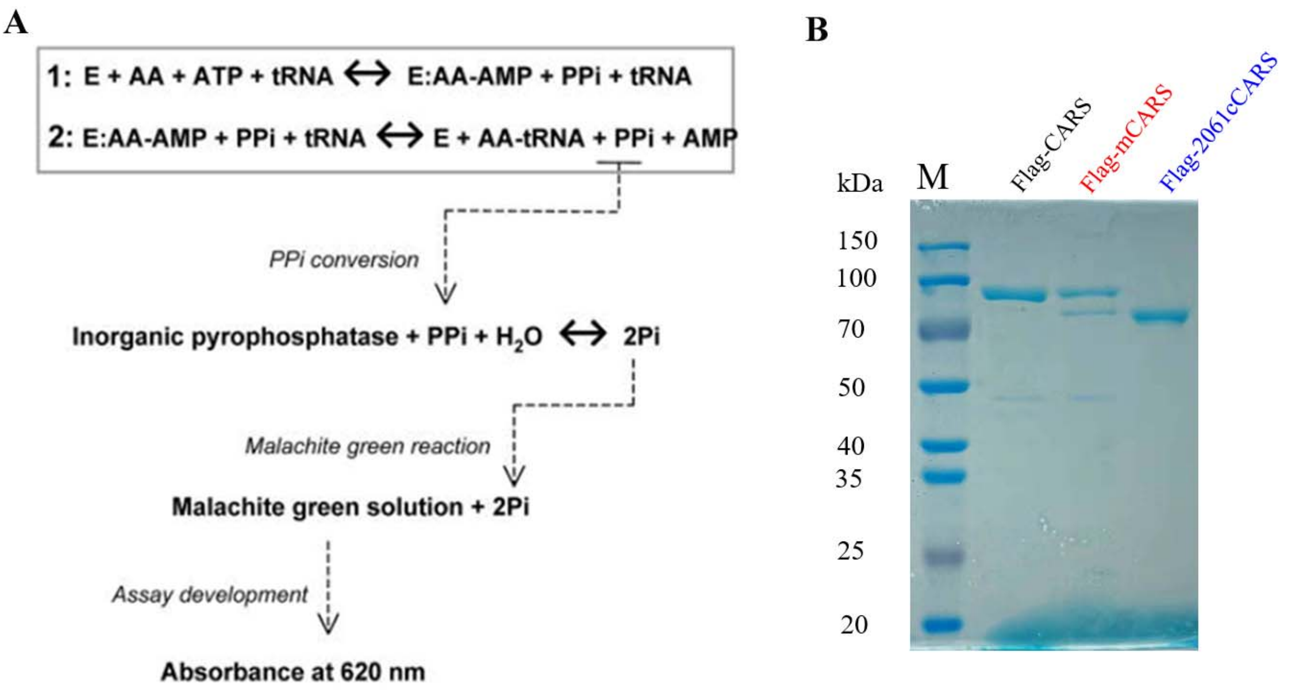

Fig. S5. Quantification of aminoacylation of *CARS* gene. A high throughput spectrophotometric assay (**A**) is used for quantitative measurement of the aminoacylation activity of three *CARS* gene sequences (**B**).
